# A diagnostic error of a necrotizing sialometaplasia: Case report

**DOI:** 10.1016/j.amsu.2021.103225

**Published:** 2022-01-03

**Authors:** Rajae El Gaouzi, Lamiae Hallab, Bouchra Taleb

**Affiliations:** Oral surgery department, Faculty of Dental Medicine-Rabat, Mohammed V University of Rabat, Morocco

**Keywords:** Necrotizing sialometaplasia, Benign lesion, Salivary glands disease, Case report

## Abstract

**Introduction:**

Necrotizing sialometaplasia (NSM) is a benign, self-limiting, inflammatory disease of salivary glands, mainly involving the minor salivary glands in the palate. This lesion can mimic a malignant neoplasm, both clinically and histopathologically, manifesting as a submucosal swelling or as an ulcer of the palate. We illustrate our work with a case of necrotizing sialometaplasia misdiagnosed as carcinoma.

**Case presentation:**

A 26 years old woman presented to dental treatment and consultation center of Rabat, for a rehabilitation of left palatal bone defect with an obturator prothesis. A postsurgical erythematous area was noted at the left palate during intraoral examination. After medical file study, we founded that she had a necrotizing sialometaplasia treated by maxillectomy of the left maxillary bone, and we realized that a diagnostic error was made leading to an aggressive treatment.

**Clinical discussion:**

Necrotizing sialometaplasia can be misdiagnosed clinically and microscopically as a malignant neoplasm, resulting in inappropriate and aggressive treatment like the case presented.

**Conclusion:**

The diagnosis of NSM is challenging, the role of histopathology and immunohistochemistry is of paramount importance.

## Highlights

• Necrotizing sialometaplasia (NSM) is a benign, self-limiting, inflammatory disease of salivary glands.
• The diagnosis of NSM is challenging, it can mimic a malignant neoplasm.
• The role of histopathological test and immunohistochemistry is of paramount importance.

## 1 Introduction

Necrotizing sialometaplasia (NSM) was first described in 1973, by Abrams et al. [1]. It's a rare, benign, self-limiting, necrotizing process involving the minor salivary glands. The incidence of NSM has been reported to account for 0.03% of all oral biopsies, but that may be underestimated because of the low recognition on this entity [2].

It is generally more frequent in men in the fourth decade of life, with a reported average age of 49 years for men and 46 years for women [3].

It most frequently develops in the palatal salivary glands, with more than 75% of all cases occurring in the posterior palate. About two-thirds of palatal cases present as unilateral and the rest are bilateral or in the midline. It has been reported in all areas where salivary gland tissue is located, including the major salivary glands, nasal cavity, sinuses, lower lip, tongue, cheek, retromolar pad, soft palate, and larynx [4].

The lesion most commonly present as an inflammatory ulcer on the mucosa of the posterior hard or soft palate. The ulcer will have indurated edges that are often raised, features strongly suggestive of a carcinoma [5]. Most necrotizing sialometaplasia cases reported a diagnosis challenge, but they didn't illustrate the treatment consequence of the diagnostic error.

The aim of this report is to present a case of necrotizing sialometaplasia misdiagnosed as carcinoma treated by maxillectomy, and to highlight the importance of histopathological and immunohistochemistry examination in the differential diagnosis of necrotizing sialometaplasia.

Below we report a case of necrotizing sialometaplasia, in line with the SCARE Criteria [6].

## 2 Case report

**Fig. 1:**
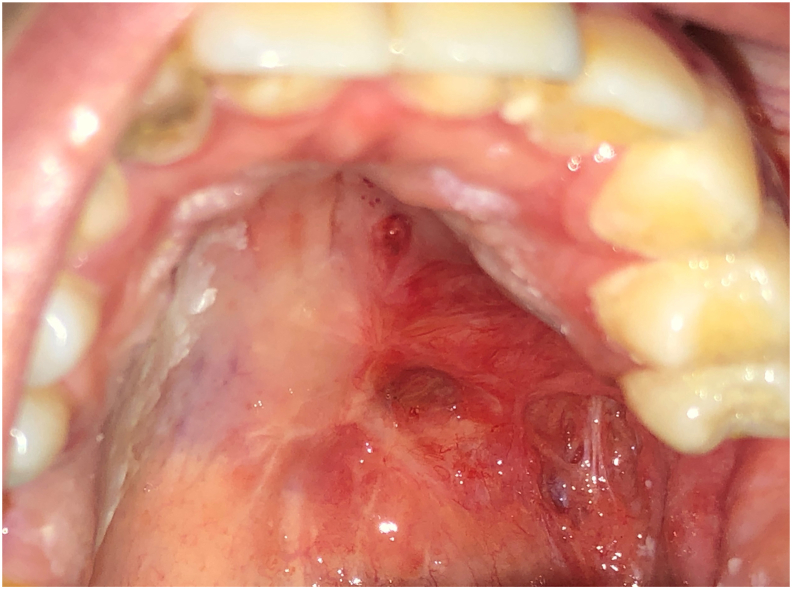
Intra-oral view after maxillectomy with an erythematous post-surgical area in the left posterior palate.

A generally healthy Caucasian 26 years old woman without toxic habits, was referred by a maxillofacial clinic to the dental treatment and consultation center of Rabat, for a rehabilitation of left palatal bone defect with an obturator prothesis. A postsurgical erythematous area was noted at the left palate during intraoral examination (Fig. 1).

**Fig. 2:**
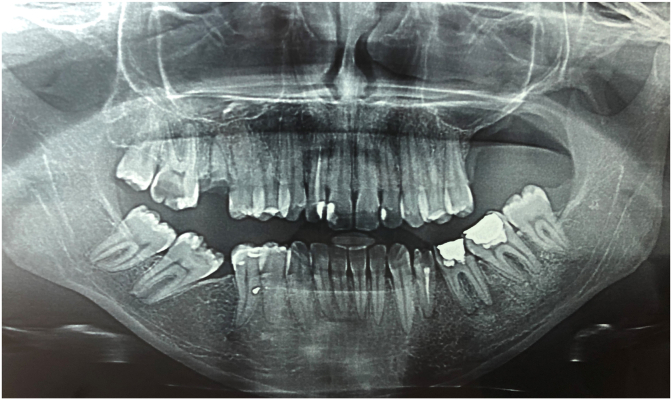
Orthopantomogram view showing maxillectomy at the left maxillary bone after surgery.

The orthopantomogram revealed a bone removal from the left maxillary bone (Fig. 2).

**Fig. 3:**
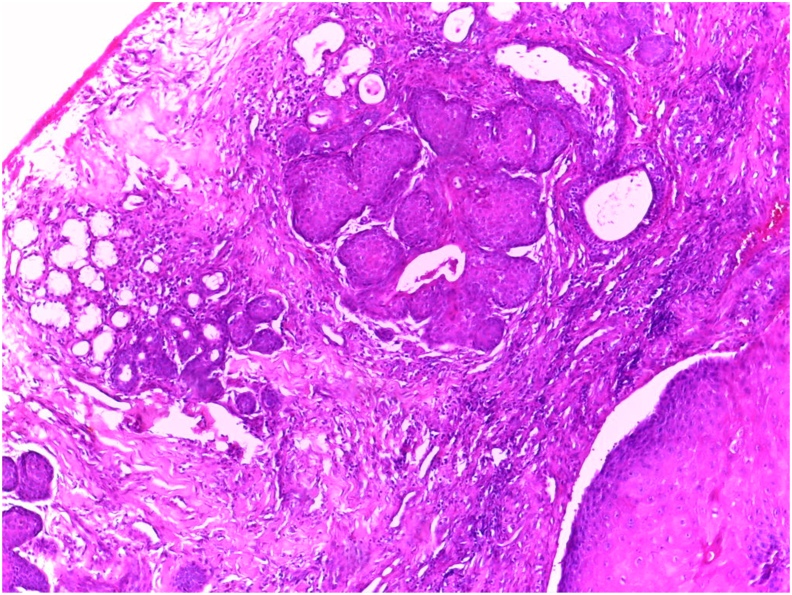
Histological photomicrograph showing a squamous metaplasia of glandular acini and salivary ducts. hematoxylin eosin [HE] stain.

**Fig. 4:**
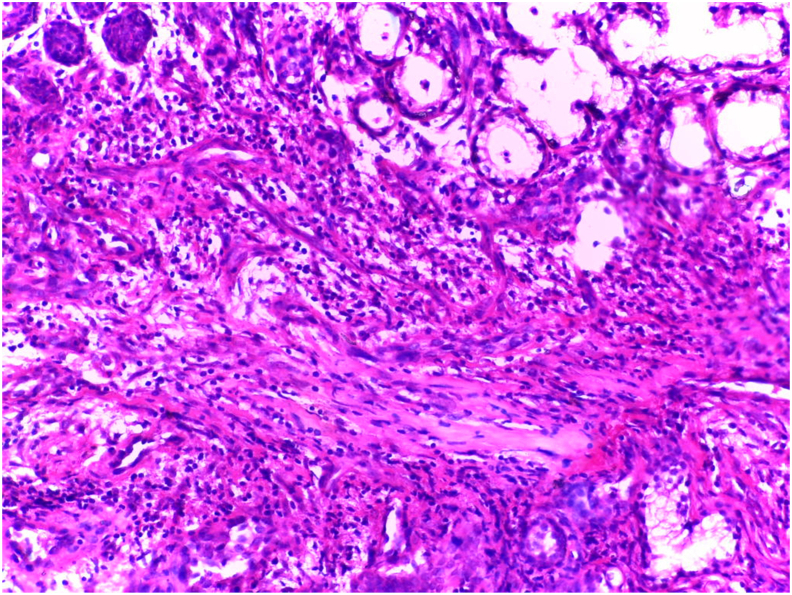
Histological photomicrograph showing an inflammatory infiltrate. hematoxylin eosin [HE] stain.

In fact, before the surgery, the patient had a slightly painful and unhealed lesion in the junction of hard and soft left palate with bone lysis, which appeared in the last two months, measuring about 2 cm and enlarges in size. The patient reports an episode of stress and anorexia. The biopsy showed an adenoid carcinoma us a result. Therefore, it was treated by maxillectomy at a maxillofacial clinic by a maxillofacial surgeon. The histopathological test of the surgical specimen revealed a necrotizing sialometaplasia, characterized by a squamous metaplasia of glandular acini and salivary ducts (Fig. 3), and an inflammatory infiltrate (Fig. 4). Two pathologists confirmed the diagnosis separately, so the diagnosis was conclusive, and it does not need immunohistochemistry examination. finally, the patient was referred by the surgeon in our hospital for a rehabilitation of left palatal bone defect with an obturator prothesis.

**Fig. 5:**
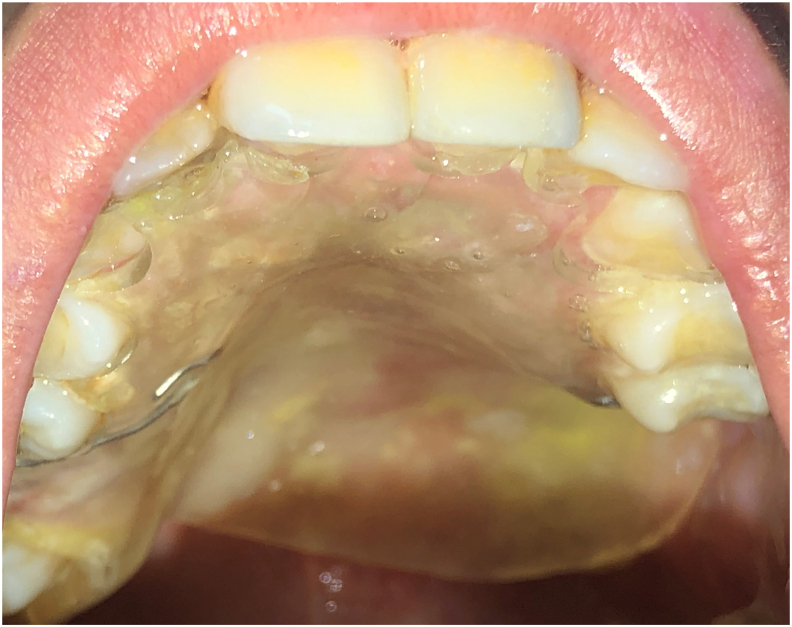
Intra-oral view of palatal tray.

After 1 month from the surgery, the obturator prothesis was placed in our dental center to protect the area for better healing (Fig. 5).

## 3 Discussion

The pathogenesis of necrotizing sialometaplasia (NSM) is unknown. Most authors sustain that some kind of physical-chemical or biological aggressions over the local blood vessels will produce ischemic changes, leading to infarction with posterior necrosis, and that NSM is the ductal proliferation in response to injury. Many causes have been suggested as possible etiologic factors including: traumatic, chemical, surgical, and infectious events including alcohol, smoking, and drug abuse. Several cases occurring in young women with anorexia and/or bulimia have been identified [7].

In other instances, some diseases characterized by vascular disturbances have been associated with NSM, supporting the theory that ischemic necrosis is the main etiologic factor [1].

The diagnosis of NSM is challenging, unfortunately, it has been misdiagnosed clinically and microscopically as a malignant neoplasm, resulting in inappropriate and aggressive treatment like the case presented. It is based on a complete clinical history and a well-oriented biopsy section [2].

Clinically, palatal ulcers with induration are strongly suggestive of malignant tumors known to occur in this area. Of these, squamous cell carcinoma is a strong consideration, as are the salivary gland malignancies, adenoid cystic carcinoma, and mucoepidermoid carcinoma. However, all of these tumors should have a longer duration than that of necrotizing sialometaplasia [5]. Other differential diagnosis of the clinical presentation included: Bacterial infection such as tuberculosis or syphilis, and granulomatous process [4].

Histopathological features of necrotizing sialometaplasia are characterized by lobular necrosis and associated squamous metaplasia of ducts and acini. At low power, the preservation of lobular architecture is apparent. The areas of necrosis consist of small pools of mucin rimmed by neutrophils. Within or adjacent to these areas are metaplastic ducts. These may be seen as ducts with a thickened squamous epithelial lining or as solid, compact, rounded nests of epithelium. An inflammatory background is typically present [5].

The mucosal surface is often ulcerated and pseudoepitheliomatous hyperplasia and squamous metaplasia of the excretory ducts are seen [8].

The pseudoepitheliomatous hyperplasia as well as the deeply seated islands of metaplastic squamous epithelium often leads to mistaken diagnosis of squamous cell carcinoma. In addition, the squamous metaplasia of the ducts and acini juxtaposed with residual mucous cells may suggest a mucoepidermoid carcinoma. However, in most cases, the lobular architecture, areas of necrosis and mixed inflammatory background, together with the distinctive epithelial nests, distinguish NS [8].

As described by Anneroth and Hansen, the histopathogenesis of NSM has five histological stages: Infarction, sequestration, ulceration, repair and healing. Histological features exhibit a spectrum ranging from ulceration, lobular necrosis, sequestration of necrotic acini, pseudoepitheliomatous hyperplasia of adjacent epithelium, squamous metaplasia of ductal epithelium and inflammatory changes [9].

As these diagnostic criteria are quite distinctive, proper care should be taken in diagnosis of this lesion, so that misdiagnosis and unnecessary radical treatment can be avoided [8].

Hematoxylin-eosin staining remains the gold standard in histopathalogical diagnosis of NSM [10].

If the diagnosis is not conclusive, it must be further supplemented via immunohistochemistry demonstrating focal to absent immunoreactivity for p53, low immunoreactivity for MIB1 (Ki-67), and the presence of 4A4/p63 and calponin-positive myoepithelial cells [10].

A true necrotizing sialometaplasia requires no specific treatment other than follow-up. However, follow-up over the subsequent 3 months is extremely important because a necrotizing sialometaplasia should resolve in that time.

The clinician should remember that incorrect diagnoses can go both ways. Just as diagnosing a necrotizing sialometaplasia as a mucoepidermoid carcinoma or a squamous cell carcinoma is possible, so is diagnosing each of these malignancies as a necrotizing sialometaplasia. Lesions that worsen over the first 2 months or show no evidence of resolving require rebiopsy and a second review of the original histopathologic features.

Necrotizing sialometaplasia fills in with granulation tissue and completely epithelializes its surface within 3 months, usually sooner [5].

## 4 Conclusion

Necrotizing sialometaplasia is giving clinical presentation of malignant neoplasm. A simple incisional biopsy is required to confirm the histological diagnosis and to rule out more serious disease processes, but histopathological specimen examination must be properly assessed before final report. Furthermore, the role of immunohistochemistry is of paramount importance.

## Ethical approval

Not applicable (Case report).

## Sources of funding for your research

None.

## Author contribution

Rajae EL GAOUZI and Lamiae HALLAB designed the concept, analyzed and interpreted the findings, wrote and reviewed the final paper under the supervision of Prof Bouchra TALEB.

## Consent

Written informed consent was obtained from the patient for publication of this case report and accompanying images. A copy of the written consent is available for review by the Editor-in-Chief of this journal on request.

## Registration of research studies

1. Name of the registry:

2. Unique Identifying number or registration ID:

3. Hyperlink to your specific registration (must be publicly accessible and will be checked):

## Guarantor

Rajae EL GAOUZI.

## Provenance and peer review

Not commissioned, externally peer-reviewed.

## Declaration of competing interest

None.
